# Tectochrysin alleviates Ang II-induced pathological cardiac hypertrophy by binding to STING and inhibiting STING/NFκB-mediated inflammation

**DOI:** 10.3389/fphar.2026.1794994

**Published:** 2026-04-23

**Authors:** Yanghao Chen, Fang Li, Ying He, Zhiyu Ling

**Affiliations:** 1 Department of Cardiology, The Second Affiliated Hospital of Chongqing Medical University, Chongqing, China; 2 Cardiovascular Neuromodulation Research and Treatment Center, Chongqing, China; 3 Department of Geriatrics, The First Affiliated Hospital of Chongqing Medical University, Chongqing, China; 4 Department of Cardiology, The Second Affiliated Hospital, School of Medicine, Zhejiang University, Hangzhou, Zhejiang, China

**Keywords:** H151, inflammation, pathological cardiac hypertrophy, STING, tectochrysin

## Abstract

**Background:**

Angiotensin II (Ang II)-induced cardiac inflammation plays a pivotal role in the pathogenesis of pathological cardiac hypertrophy and hypertension-related heart failure. Tectochrysin (Tec) is a flavonoid natural compound exhibiting significant anti-inflammatory activity. However, the role of Tec in hypertensive heart failure and its molecular targets remain unclear.

**Methods:**

The therapeutic efficacy of Tec was assessed in the Ang II-induced mouse model using echocardiography, histopathological staining, and serological tests. Its anti-hypertrophic effect was further examined *in vitro* by phalloidin staining. Investigate the mechanism of action of Tec through transcriptome sequencing. The interaction of Tec with STING was detected by DARTS, CETSA, and SPR assays. Western blotting assay detected the effect of Tec on Ang II-induced activation of the STING/NFκB pathway. The functional dependency of Tec on STING was demonstrated using the STING inhibitor H151.

**Results:**

*In vivo* experiments confirmed that Tec alleviates Ang II-induced myocardial inflammation, pathological hypertrophy, myocardial fibrosis, and cardiac dysfunction. Further *in vitro* studies revealed the efficacy of Tec in inhibiting cardiomyocyte hypertrophy. Mechanistically, Tec significantly suppressed Ang II-induced activation of the STING/NFκB signalling pathway by targeting STING. Crucially, administration of the STING inhibitor H151 alleviates pathological myocardial hypertrophy, however its use diminishes the therapeutic effect of Tec.

**Conclusion:**

Our findings confirmed that Tec may represent a promising lead compound targeting STING for pathological cardiac hypertrophy.

## Graphical Abstract

**Figure:**
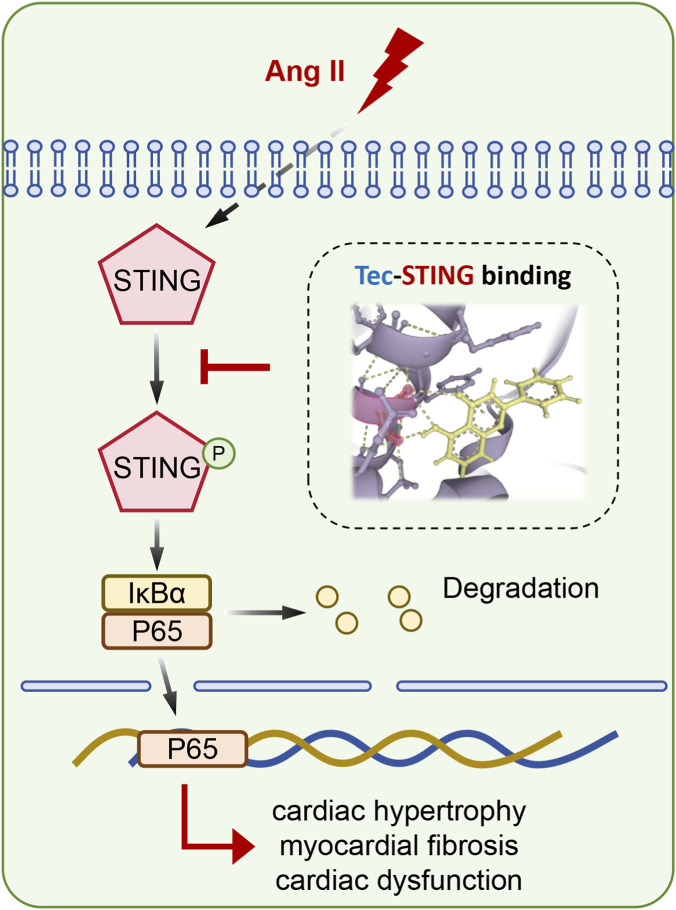

## 1 Introduction

Pathological cardiac hypertrophy, as an increasingly serious global public health issue, is closely associated with multiple cardiovascular diseases, particularly heart failure (Dong et al., 2024; Julie M T et al., 2025). The development of heart failure are closely associated with excessive activation of the renin-angiotensin system (RAS), with angiotensin II (Ang II) being one of the most important mediators involved (Noemi et al., 2021; Vinicius et al., 2021). Although inhibitors of the RAS, such as angiotensin-converting enzyme (ACE) inhibitors or angiotensin receptor blockers (ARBs), have been proven to mitigate ventricular remodeling and myocardial fibrosis by blocking the RAS axis (Dayue Darrel, 2015; Jingsi, Lina and Yanchun, 2021; Youming et al., 2021), often cannot fully prevent progression (Lauren B et al., 2019). Consequently, the discovery and development of novel agents against pathological cardiac hypertrophy are urgently required.

Persistent activation of chronic inflammation and innate immunity constitutes a key pathological mechanism underlying the onset and progression of pathological cardiac hypertrophy and heart failure (Laura et al., 2021; Samar et al., 2025; Wenke et al., 2025). Notably, numerous studies have shown that continuous infusion of Ang II can trigger a significant inflammatory response in heart (Dong et al., 2024; Yu et al., 2025). Therefore, anti-inflammatory therapy may represent an effective strategy for treating Ang II-induced pathological cardiac hypertrophy. The STING-NFκB signalling pathway is a classical signalling pathway involved in inflammation and innate immunity (Cailan, Xinyi, Shichun, Qiaoyuan and Shixiong, 2025; Weihong et al., 2025; Ziyang et al., 2024). In recent years, the STING-NFκB signaling pathway has been identified as a critical regulator in pathological cardiac hypertrophy. It has been confirmed that knockout or pharmacological inhibition of STING alleviates pathological cardiac hypertrophy by suppressing the downstream NFκB pathway, whereas cardiomyocyte-specific activation of STING exacerbates its development (Lintao et al., 2023). Additionally, another study found that RNF5 inhibits pathological hypertrophy by promoting STING degradation, which subsequently suppresses IκBα degradation and P65 phosphorylation (Lu-Lu et al., 2022). Therefore, targeting and inhibiting the STING/NFκB pathway represents a viable therapeutic approach in the management of Ang II-induced pathological cardiac hypertrophy.

Traditional Chinese medicine has yielded satisfactory results compared to chemically synthesised antihypertensive drugs (Mengyang et al., 2022; Na et al., 2022; Zimin et al., 2024). Tectochrysin (Tec), a flavonoid compound, can be isolated from *propolis*, *Alpinia oxyphylla Miq*, and *Lychnophora markgravii* (Lei et al., 2021). Researchers have discovered that Tec exhibits favourable anti-inflammatory activity. Tec has been reported to inhibit LPS-induced macrophage inflammation by suppressing activation of the MEK1/2-ERK1/2 axis (Rui et al., 2017). Another study showed that Tec suppresses inflammatory activation by targeting the macrophage JAK3/STAT3 signalling pathway, thereby improving rheumatoid arthritis (Pin, Wei, Shuyi, Lianying, & Jianjun, 2025). It is worth noting that Tec has also been reported to possess cardioprotective effects. In vanadium-induced cardiac injury, Tec modulates inflammation and apoptosis by regulating the NLRP3, JAK1/STAT3, and NFκB pathways (Yahui et al., 2025). Based on the aforementioned findings, we hypothesise that Tec may emerge as a potential natural therapeutic agent for Ang II-induced pathological cardiac hypertrophy.

This study aimed to investigate the therapeutic efficacy of Tec in Ang II-induced pathological cardiac hypertrophy, along with its molecular mechanisms and action targets. We showed that Tec significantly alleviated Ang II-induced cardiac dysfunction, myocardial inflammation, myocardial fibrosis, and pathological cardiac hypertrophy. Notably, subsequent molecular mechanism studies revealed that Tec exhibits unique anti-inflammatory activity by directly acting on STING and inhibiting the NFκB-mediated inflammatory pathway downstream of STING. Crucially, the aforementioned inhibition of the NFκB pathway by Tec was no longer significant following administration of the STING inhibitor H151. Based on these findings, Tec demonstrates potential as a novel therapeutic agent targeting STING for the treatment of hypertensive cardiac hypertrophy.

## 2 Methods and materials

### 2.1 Reagents

Ang II (HY-13948), and H151 (HY-112693) were purchased from MedChemExpress (New Jersey, USA). Tec (520–28–5, purity of 99.8%) were purchased from Alfa Biotechnology (Chengdu, China). GAPDH (ET1601-4) were obtained from HUABIO (Hangzhou, China). IκBα (9242), p-STING (50,907) were obtained from Cell Signaling Technology (MA, USA). P65 (10745-1-AP), STING (19851-1-Ap), Lamin B (12987-1-AP) were obtained from Proteintech (Hubei, China). Plasmids (Flag-STING, Flag-cGAS, Flag-STING-S161A) were obtained from GenScript (Nanjin, China). Cells were transfected with overexpression plasmids using Lipofectamine 3000 and P3000 (Invitrogen, USA).

### 2.2 Animal experiments

Given that female mice exhibit a potential protective effect against myocardial fibrosis due to higher oestrogen levels (Lejla, Laila and Mansoureh, 2019), this may interfere with the establishment of disease models. To achieve significant cardiac injury under Ang II induction, we referenced strategies for mouse sex selection from published studies on hypertensive pathological hypertrophy (Bozhi et al., 2023; Xue et al., 2025). Consequently, all *in vivo* experiments in this study were conducted in male mice to evaluate the therapeutic effects of Tec and its underlying mechanisms. 8-week-old male C57BL/6 J mice were procured from Huachuang China Pharmaceutical Technology Co., Ltd. (Jiangsu, China). All animals were housed under specific pathogen-free conditions with controlled environmental parameters (temperature: 20 °C–25 °C; humidity: 50% ± 5%; standard 12/12-h light/dark cycle). Mice had free access to sterile water and chow. Based on prior studies, pathological cardiac hypertrophy was induced by continuous 4-week infusion of angiotensin II (1 μg/kg/min) or saline (six mice per group) via osmotic pumps (1004W, RWD Life Science, Nanjin, China). According to previous research reports (Bozhi et al., 2023), osmotic pumps were implanted subcutaneously in the dorsal region of each mouse. In the Tec efficacy assessment experiment, mice were infused with Ang II for 4 weeks and treated with Tec (2.5 mg/kg or 5 mg/kg, i. p., qd) for the last 2 weeks of the infusion period. The dosage of Tec was based on previous research (Lei et al., 2021). To elucidate the pivotal role of STING in Tec therapy for pathological cardiac hypertrophy in vivo experiments, Ang II-induced mice were treated with STING inhibitor H151 (4 μg/kg i. p., qd) for 4 weeks. From the third week onwards, daily intraperitoneal injections of Tec (5 mg/kg, i. p., qd) were administered. The dosage of H151 was determined by reference to previous studie (Thuy Linh et al., 2025).

### 2.3 Echocardiography

Mice were anaesthetised with 2% isoflurane and placed on an echocardiography board. Once heart rate and respiration stabilised, M-mode images and echocardiographic parameters were acquired using a multimodal ultrasound imaging system (Vevo 770, Visual Sonics, Canada).

### 2.4 Biochemical assay

Serum ANP, IL-1β, IL-6, and TNF-α were measured using commercial kits (Fine Biotech, Hubei, China). Serum ALT, AST, creatinine, and BUN kits were obotained from Rayto Life Sciences (Shenzhen, China). All the experiments were performed according to the manufacturer’s instructions.

### 2.5 Cell extraction and culture

According to previous research (Bozhi et al., 2023), we isolated primary cardiomyocytes. In brief, rats within 3 days of birth were euthanised, their hearts excised and washed with PBS to remove blood. The heart tissue was subsequently digested using a mixture of trypsin and collagenase, and primary cardiomyocytes were isolated from fibroblasts via differential adhesion culture. We induced hypertrophy in primary cardiomyocytes by treating them with 1 μM Ang II for 48 h. The NIH/3T3 cells were procured from the Shanghai Institute of Biochemistry and Cell Biology (Shanghai, China). We cultured the cells using DMEM medium supplemented with 10% foetal bovine serum, 1% penicillin/streptomycin, and 4.5 g/L glucose, and placed the cells in a humidified incubator maintained at 37 °C with 5% carbon dioxide. To downregulate the expression of STING, we employed LipofectAMINE 3000 (Cat# L3000015; Invitrogen, United States) to transfect cells with siSTING (Sequence: AGU​UUG​AAG​CGG​UAA​CUU​CUG). The negative control was prepared using scrambled sequences.

### 2.6 TRITC-phalloidin staining

Primary cardiomyocytes were seeded onto glass-bottomed culture dishes and processed according to the experimental protocol. The medium was subsequently removed, and cells were washed three times with PBS buffer. They were then fixed at room temperature with 4% paraformaldehyde solution for 15 min, followed by three PBS washes. Cells were treated with 0.5% Triton X-100 solution for 10 min, followed by three PBS washes. Incubate cardiomyocytes with TRITC-phalloidin working solution (Catalog No. CA1610; Solarbio, Beijing, China) at room temperature for 30 min under light-protected conditions. Finally, stain nuclei with DAPI-containing anti-fluorescence quencher, observe under fluorescence microscopy, and record results.

### 2.7 Histological analysis

For histological staining of the samples, we first fixed the cardiac tissue in 4% paraformaldehyde, followed by dehydration and paraffin embedding. Following the manufacturer’s protocol, pre-prepared 5 μm-thick cardiac tissue sections were sequentially stained with H&E staining (G1120, Solarbio Life Sciences, Beijing, China), Masson’s trichrome (G1340, Solarbio Life Sciences, Beijing, China), WGA-FITC staining (GTX01502, GeneTex Corporation, Texas, USA), and Sirius Red staining (S8060, Solarbio Life Sciences, Beijing, China). For immunohistochemistry, sections were deparaffinized and subjected to antigen retrieval. The sections were then incubated in 3% H_2_O_2_ for 30 min at room temperature in the dark to block endogenous peroxidase activity. After incubation with the appropriate primary and secondary antibodies, immunoreactivity was visualized using DAB reagent. Stained sections were observed and photographed under a microscope.

### 2.8 Western blot assay

Protein samples were lysed using RIPA buffer. Equal volumes of protein samples were separated by sodium dodecyl sulphate-polyacrylamide gel electrophoresis at 80 V. Proteins were clearly resolved, and the gel proteins were transferred to a PVDF membrane at 300 mA. After blocking with 5% BSA at room temperature for 1 hour, the membrane was incubated overnight with the primary antibody at 4 °C. Following thorough incubation, the bands treated with TBST were incubated with HRP-labelled secondary antibody at room temperature for 1 hour. Results were finally detected and recorded using the ECL system.

### 2.9 RNA sequencing

To further investigate the specific mechanism by which Tec exerts its effects, we selected male mice samples from Ang II + Tec5 group and Ang II group for mRNA sequencing analysis (n = 3 per group). The sequencing depth for the transcriptome is 200X. Sequencing was performed using the Illumina HiSeq 2500 platform. First, Cutadapt was applied to remove reads containing adapter contamination, low-quality bases, and unidentified bases. The specific quality control standards are as follows: (1) removing reads containing adapters; (2) removing reads containing polyA and polyG; (3) removing reads containing more than 5% of unknown nucleotides (N); (4) removing low quality reads containing more than 20% of low quality (Q-value≤20) bases. Subsequently, FastQC was employed to validate sequence quality. Finally, bioinformatics analysis was performed.

### 2.10 Tec-STING docking assay

The structural data for Tec (CID: 5281954) is sourced from PubChem (https://pubchem.ncbi.nlm.nih.gov/). The crystal structure data for the STING protein (PDB ID:4KC0) is sourced from the PDB database (http://www.rcsb.org/pdb/). We employed the online docking platform (https://www.dockeasy.cn/) to perform docking between the Tec and STING.

### 2.11 Drug affinity response target stability (DARTS) assay

Based on the principle that small-molecule drugs enhance protein structural stability by specifically binding to target proteins to alter their conformation or mask cleavage sites, the dart technology has been widely applied in detecting the binding of small-molecule drugs to targets (Brett, Gwanghyun, James A, and Jing, 2012; Qi-Hao et al., 2026; Qingxiu et al., 2026). Therefore, we employed the DARTS method to detect the binding of Tec to potential target proteins. We first collected protein samples by centrifugation and determined protein concentration. Subsequently, the lysates were incubated overnight at 4 °C with 5 μM Tec. Next, pronase (at a concentration of 25 ng per 1 μg total cellular protein) was added to the lysates and incubated at room temperature for 1 hour. All samples were loaded into loading buffer and boiled for 10 min before undergoing Western blot analysis to detect STING expression levels.

### 2.12 Cellular thermal shift assay (CETSA)

Based on the principle that small molecules can alter protein thermal stability upon binding, we conducted CETSA. This assay has been widely employed in the screening and validation of small molecule drug targets (Hong-Lei, Huan, Xiao-Yan, Tian-Rui and Xiao-Xi, 2026; Mengyao et al., 2026; Yangping et al., 2026). Samples were treated with 5 μM Tec or DMSO for 6 h, followed by lysis using RIPA lysis buffer. The total sample was equally divided into 8 groups. All groups except the control underwent heat treatment by heating for 5 min at 37, 43, 48, 53, 58, 63, and 69 °C, respectively. Samples were cooled to room temperature within 3 min and centrifuged at 12,000 r/min for 15 min. The supernatant was mixed with loading buffer and heated at 100 °C for 10 min. Western blot analysis was subsequently performed according to the previously described protocol.

### 2.13 Surface plasmon resonance (SPR) assay

To validate Tec-STING binding via SPR assay, we constructed a recombinant STING protein. Concurrently, Tec was diluted to six concentrations (200, 100, 50, 25, 12.5 and 0 μM). The prepared STING protein solution was injected into the pre-activated chip. Subsequently, each Tec concentration was sequentially introduced into the channel at a fixed flow rate to bind with the chip, with the sensor chip recording the data.

### 2.14 Quantitative polymerase chain reaction (qPCR)

Total RNA was extracted from samples using a SteadyPure Universal RNA Extraction Kit (AG21022, Accurate Biology, China). The extracted RNA was then reverse-transcribed into complementary DNA (cDNA) with an Evo M-MLV RT-PCR Kit (AG11728, Accurate Biology, China). The qPCR was performed using SYBR Green reagent kits (AG11701, Accurate Biology, China) under the cycling conditions detailed in Supplementary Table S4. The primer sequences employed for qPCR are listed in Supplementary Table S1.

### 2.15 Data analysis

The experimental data presented herein are expressed as the mean ± standard error of the mean (SEM). In order to ascertain significant differences between two groups, the Student’s t-test was employed. In instances where the comparison involved more than two groups, a one-way analysis of variance (ANOVA) was conducted, followed by a Tukey *post hoc* test. All statistical analyses were performed using GraphPad Prism 8.0 software, with statistical significance set at p < 0.05.

## 3 Results

### 3.1 Tec alleviates Ang II-induced cardiac dysfunction and left ventricular hypertrophy in mice

**FIGURE 1:**
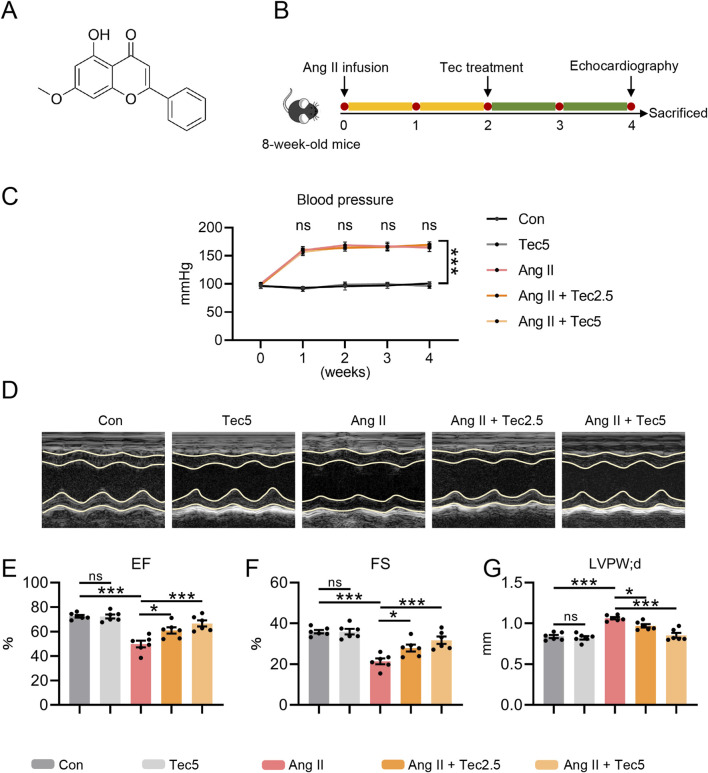
Tec alleviates Ang II-induced cardiac dysfunction and left ventricular hypertrophy in mice **(A)** Structural formula of Tec. **(B)** Schematic diagram of the *in vivo* experimental procedure. Mice underwent 4 weeks of Ang II treatment. At the start of the third week, mice received Tec administration (2.5, 5 mg/kg, i. p.). **(C)** Blood pressure measurement results for each group of mice. **(D)** Representative echocardiographic images for each group of mice. **(E)** EF in the indicated groups. **(F)** FS in the indicated groups. **(G)** Left ventricular posterior wall thickness at diastole of mice. N = 6. ns, p > 0.05. *, p < 0.05. **, p < 0.01. ***, p < 0.001.

The structural formula of Tec is shown in Figure 1A. To preliminarily assess the effect of Tec on cardiac hypertrophy, we first applied two therapeutic concentrations of Tec to Ang II-induced mice (Figure 1B). We observed that Ang II infusion increased systolic blood pressure, which was not reversed by Tec treatment, indicating no effect of Tec on blood pressure (Figure 1C). However, echocardiographic findings revealed that Tec treatment significantly ameliorated Ang II-induced cardiac dysfunction, as evidenced by improvements in EF and FS (Figures 1D–F). Furthermore, we observed that Ang II treatment resulted in significant thickening of the diastolic left ventricular posterior wall in mice, whereas Tec administration markedly attenuated this pathological manifestation (Figure 1G). Collectively, these results demonstrate that Tec treatment markedly alleviates Ang II-induced cardiac dysfunction and left ventricular hypertrophy.

### 3.2 Tec ameliorates Ang II-induced pathological cardiac hypertrophy and myocardial fibrosis in mice

**FIGURE 2:**
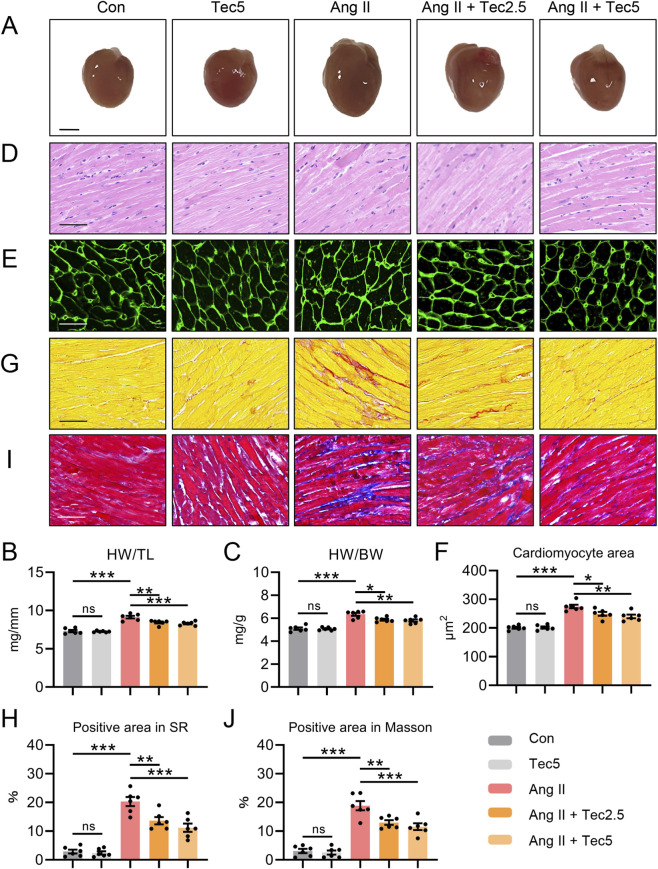
Tec ameliorates Ang II-induced pathological cardiac hypertrophy and myocardial fibrosis in mice **(A)** Gross image of the heart. Scale bar = 2 mm. **(B)** HW/TL ratio in the indicated groups. **(C)** HW/BW ratio in the indicated groups. **(D)** The representative H&E results in the indicated groupss. Scale bar = 50 μm. **(E)** The representative image of WGA results. Scale bar = 25 μm. **(F)** Quantitative analysis of panel **(E) (G)** The representative Sirius red staining results. Scale bar = 50 μm. **(H)** Quantitative analysis of panel **(G) (I)** The representative Masson staining results. Scale bar = 50 μm. **(J)** Quantitative analysis of panel **(I)** N = 6. ns, p > 0.05. *, p < 0.05. **, p < 0.01. ***, p < 0.001.

Ang II frequently induces pathological cardiac hypertrophy and fibrosis alongside cardiac dysfunction. Consequently, macroscopic examination of mouse hearts revealed that Tec significantly ameliorated Ang II-induced cardiac enlargement (Figure 2A). Similarly, analogous outcomes were observed for the heart weight/tibia length ratio (HW/TL) and heart weight/body weight ratio (HW/BW) (Figures 2B,C). To further evaluate antihypertrophic effects of Tec, we performed haematoxylin and eosin (H&E) staining on cardiac tissues from each group. As anticipated, Ang II treatment resulted in markedly thickened myocardial fibres with disorganised arrangement, whereas Tec treatment led to significantly thinner fibres with more orderly alignment (Figure 2D). Furthermore, WGA staining results revealed that Tec treatment significantly attenuated Ang II-induced cardiac hypertrophy compared to the Ang II group (Figures 2E,F). We further examined the transcriptional levels of Myh7, Anp, and Bnp in myocardial tissue. Consistent with prior pathological findings, PCR results demonstrated that Tec significantly suppressed Ang II-induced elevations in the mRNA level of Myh7, Anp, and Bnp levels (Supplementary Figure S1A–C). These results indicate that Tec alleviates Ang II-induced pathological myocardial hypertrophy. To thoroughly evaluate the effects of Tec on Ang II-induced cardiomyocyte hypertrophy, we first screened for suitable *in vitro* drug concentrations using the CCK8 assay. We observed that Tec concentrations of 10 μM and above adversely affected cardiomyocyte viability (Supplementary Figure S2). Consequently, we selected 5 μM Tec for the *in vitro* experiments. The phalloidin staining revealed that Ang II treatment significantly exacerbated cardiomyocyte hypertrophy, whereas co-treatment with Tec markedly alleviated Ang II-induced hypertrophic phenotype in cardiomyocytes (Supplementary Figures S3A,B). Similarly, we examined the transcriptional levels of Myh7, Anp, and Bnp in cardiomyocytes. PCR results demonstrated that Tec significantly suppressed the Ang II-induced elevation of Myh7, Anp, and Bnp levels in cardiomyocytes, further indicating that Tec can alleviate Ang II-induced cardiomyocyte hypertrophy (Supplementary Figures S4A–C). Subsequently, we employed Sirius red staining of mouse cardiac tissue to evaluate the effect of Tec on Ang II-induced myocardial fibrosis. Sirius red results indicated that Tec significantly reduced fibrillar deposition in the heart tissue of Ang II mice (Figures 2G,H). Moreover, similar findings were observed in Masson staining results (Figures 2I,J) and immunohistochemical staining of Col-1 (Supplementary Figures S5A,B), suggesting that Tec exerts anti-fibrotic effects in the Ang II-induced mice. Notably, compared to controls, Tec treatment alone did not significantly alter myocardial tissue morphology or fibrosis levels, indicating that Tec therapy possesses favourable safety. Furthermore, Tec treatment alone did not affect mouse body weight, kidney weight, liver weight, or serum levels of ALT, AST, urea nitrogen, and creatinine (Supplementary Figures S6A–G). Collectively, these results indicate Tec treatment markedly ameliorates Ang II-induced pathological cardiac hypertrophy and myocardial fibrosis.

### 3.3 Tec improves Ang II-induced myocardial injury and myocardial inflammation

**FIGURE 3:**
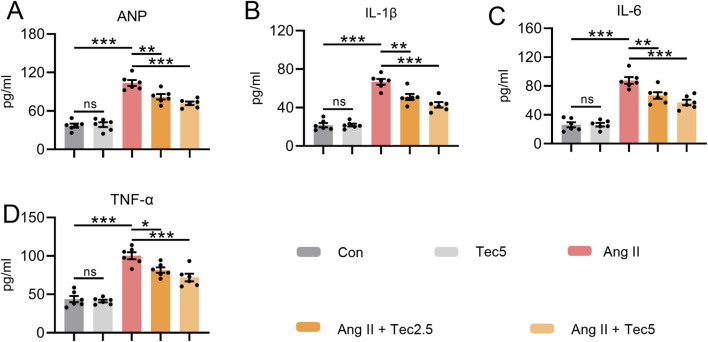
Tec improves Ang II-induced myocardial injury and myocardial inflammation Mice received Ang II infusion over a 4-week period, with Tec treatment administered concurrently during the final 2 weeks. **(A)** ANP levels in mice serum. **(B)** IL-1β levels in serum. **(C)** IL-6 levels in serum. **(D)** TNF-α levels in serum. N = 6. ns, p > 0.05. *, p < 0.05. **, p < 0.01. ***, p < 0.001.

Ang II infusion frequently induces myocardial injury and chronic inflammation. Therefore, we further investigated the effects of Tec on Ang II-induced myocardial injury and myocardial inflammation levels. First, serum samples were collected from each group for ANP detection. As anticipated, Ang II treatment elevated serum ANP levels. However, following Tec administration, ANP levels in the treated group of mice decreased significantly in a dose-dependent manner (Figure 3A). These findings further demonstrate that Tec mitigates the severity of Ang II-induced heart failure and myocardial injury in mice. Additionally, we measured serum IL-1β levels, revealing that Tec significantly reduced serum IL-1β levels in mice following Ang II induction. (Figure 3B). Similar findings were observed in serum IL-6 (Figure 3C) and TNF-α (Figure 3D) assays. We also examined the level of macrophage infiltration in myocardial tissue, finding that Ang II treatment led to increased macrophage infiltration, whereas Tec treatment suppressed this pathological process (Supplementary Figures S7A,B). Collectively, these data demonstrate that Tec significantly ameliorates Ang II-induced myocardial injury and inflammation.

### 3.4 Tec can bind to STING via the Ser161 of STING

**FIGURE 4:**
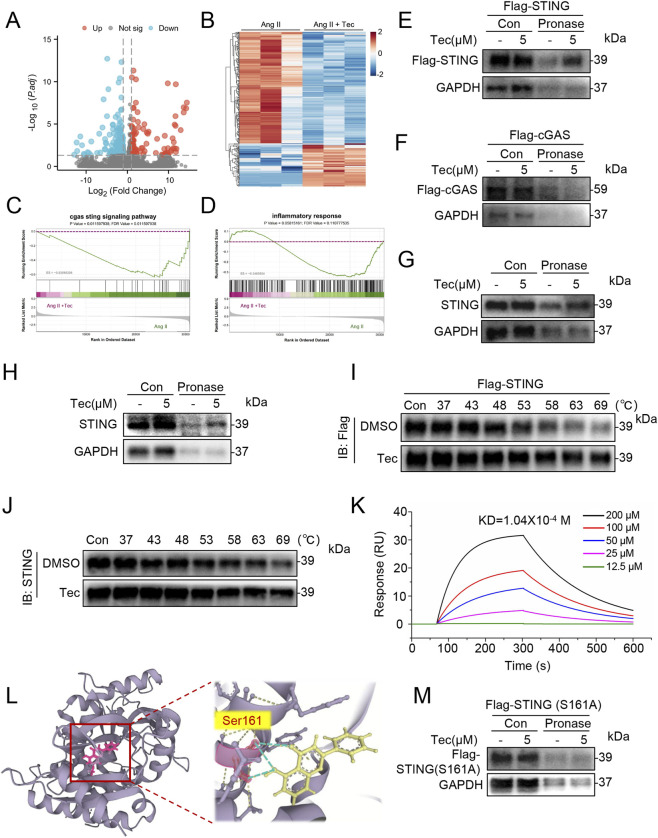
Tec can bind to STING via the Ser161 of STING. **(A)** Volcano plot analysis results of the RNA sequencing. **(B)** Heatmap analysis results of the RNA sequencing. **(C)** GSEA analysis of the “cgas sting signaling pathway” in the comparison between the Ang II and Ang II + Tec groups. **(D)** GSEA analysis of the “inflammatory response” in the comparison between the Ang II and Ang II + Tec groups. **(E)** Following transfection of NIH/3T3 cells with Flag-STING for 24 h, the collected lysates were subjected to DARTS analysis. **(F)** Following transfection of Flag-cGAS for 24 h, the NIH/3T3 cells were subjected to DARTS analysis. **(G)** Primary cardiomyocytes were stimulated with Ang II for 48 h, after which lysates were collected for DARTS analysis. **(H)** DARTS experiment using heart tissue treated with Ang II for 4 weeks. **(I)** Following transfection with Flag-STING for 24 h, the NIH/3T3 cells were subjected to CETSA analysis. **(J)** Primary cardiomyocytes were treated with Ang II for 48 h, followed by CETSA analysis to detect STING-Tec interactions. **(K)** SPR analysis results for Tec and STING. **(L)** Molecular docking results for Tec and STING. **(M)** Following transfection with Flag-STING-S161A for 24 h, the NIH/3T3 cells were subjected to DARTS analysis.

To further investigate the mechanism by which Tec exerts its cardioprotective effects, we first performed RNA sequencing on cardiac tissue from Ang II and Ang II + Tec groups, followed by volcano plot analysis (Figure 4A) and heatmap analysis (Figure 4B). Results indicated that, compared with the Ang II group, the Ang II + Tec group exhibited marked alterations in the gene expression profile. More importantly, GSEA revealed that the cGAS-STING signaling pathway ranked among the top ten most significantly altered pathways and was markedly downregulated following Tec treatment (Figure 4C; Supplementary Table S2). Meanwhile, the “inflammatory response” gene set was also downregulated (Figure 4D). Notably, recent studies have reported that cGAS/STING pathway can mediate cardiac inflammation and thereby contribute to the development of pathological cardiac hypertrophy (Cheng et al., 2025; Dan et al., 2020; Patrick Kwabena et al., 2022). Thus, we speculated that Tec acts through either cGAS or STING to regulate myocardial inflammation and pathological cardiac hypertrophy. We then applied the DARTS assay, a proven method for identifying drug target proteins. Based on previously reported literature (Bozhi et al., 2023; Jibo et al., 2024), NIH/3T3 cells, a commonly used tool cell line in cardiovascular research, were selected for exogenous experiments in this study. Subsequently, STING and cGAS were individually overexpressed via plasmid transfection. Western blot analysis revealed that Tec treatment significantly increased the pronase resistance of the STING protein (Figure 4E; Supplementary Figure S8A), but failed to alter cGAS pronase resistance (Figure 4F; Supplementary Figure S8B). This specific stabilization preliminarily suggested that STING, rather than cGAS, might be the direct binding target of Tec. To validate this potential interaction, we repeated the DARTS assay in Ang II-induced primary cardiomyocytes. Consistent with our hypothesis, pronase-treated samples in the Tec group retained significantly higher STING expression compared to the control group (Figure 4G). We further collected Ang II-induced mouse cardiac tissue for DARTS analysis. Consistent with our hypothesis, Tec administration enhanced pronase resistance of STING (Figure 4H). Also, exogenous CETSA analysis indicates that Tec significantly enhances the thermal stability of exogenous STING, thereby delaying its degradation process (Figure 4I; Supplementary Figure S8C). Similarly, we observed this phenomenon in Ang II-induced heart tissue samples (Figure 4J). To confirm direct interaction between Tec and STING, we employed SPR detection, which indicated the binding affinity is 1.04 × 10^−4^ M, suggesting a stable interaction between Tec and STING (Figure 4K). Moreover, drug-protein docking analysis with the highest binding energy result indicated that Tec likely interacts with STING via its serine residue at position 161 (Figure 4L). DARTS experiments revealed that when STING Ser161 was mutated to alanine, Tec no longer significantly affected STING protein stability (Figure 4M). Furthermore, we conducted CETSA experiments using the STING-S161A plasmid. The results demonstrated that when the serine at position 161 of STING was mutated to alanine, the effect of Tec in enhancing the thermal stability of the STING protein was no longer significant (Supplementary Figure S8D,E). In summary, Tec can bind to STING via the Ser161 of STING.

### 3.5 Tec inhibits Ang II-induced STING phosphorylation and reduce the activation of its downstream NFκB pathway

**FIGURE 5:**
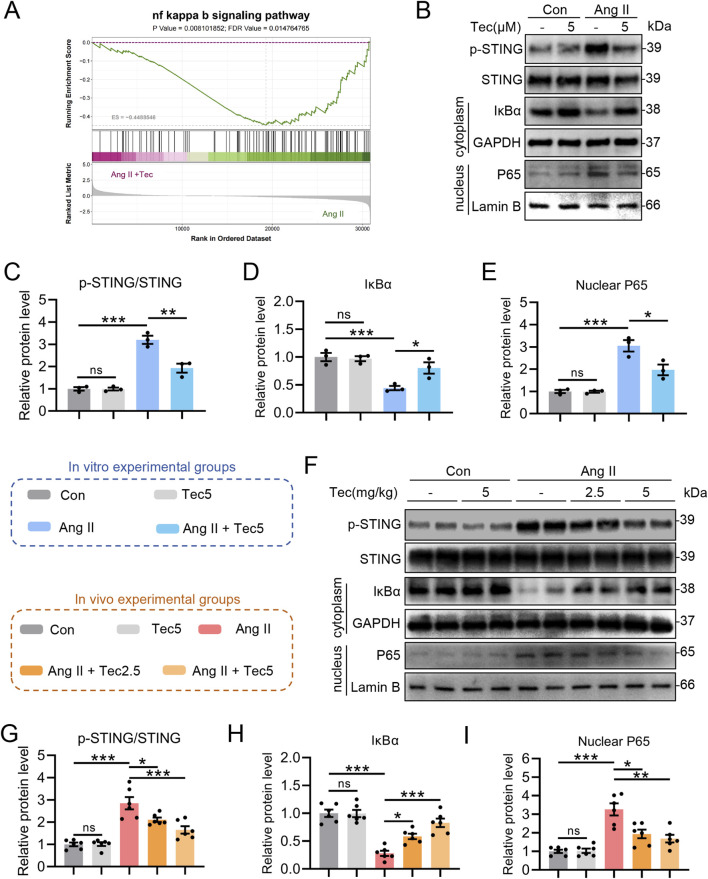
Tec inhibits Ang II-induced STING phosphorylation and reduce the activation of its downstream NFκB pathway. **(A)** GSEA analysis of the NFκB pathway in the comparison between the Ang II and Ang II + Tec groups. **(B)** After pretreatment with Tec for 1 h, the primary cardiomyocytes were stimulated with Ang II for 48 h. Representative immunoblotting results for the STING-NFκB pathway. **(C)** Quantitative analysis of phosphorylated STING. **(D)** Quantitative analysis of IκBα. **(E)** Quantitative analysis of nuclear P65. **(F)** Representative immunoblotting results for the STING-NFκB pathway in the heart tissue of mice. **(G)** Quantitative analysis of phosphorylated STING in heart tissue. **(H)** Quantitative analysis of IκBα in heart tissue. **(I)** Quantitative analysis of nuclear P65 in heart tissue. For *in vitro* experiments, N = 3. For the *in vivo* experiments, N = 6. ns, p > 0.05. *, p < 0.05. **, p < 0.01. ***, p < 0.001.

To investigate the downstream mechanisms after Tec targets STING, we performed GSEA on inflammation-related pathways, which revealed that Tec treatment significantly suppressed the NFκB pathway (Figure 5A). As an upstream regulator of the NFκB pathway, STING promotes pathway activity through its phosphorylation and activation, which leads to the development of cardiac inflammation and hypertrophy (Lintao et al., 2023; Zimin et al., 2024). Consequently, we proceeded to evaluate the effect of Tec on STING-NFκB pathway. First, we applied Tec to cardiomyocytes and assessed STING phosphorylation, IκBα degradation, and P65 nuclear translocation. The results showed that Tec treatment significantly suppresses Ang II-induced activation of the STING/NFκB pathway in cardiomyocytes (Figures 5B–E). Concurrently, we examined expression levels of STING/NFκB signalling pathway-associated proteins in animal tissues. Consistently, we observed significant suppression of STING phosphorylation and NFκB pathway activation in the Tec + Ang II group compared to the Ang II group (Figures 5F–I). Furthermore, the changes in STING/NFκB pathway-related genes observed in the sequencing data (Ang II + Tec group vs. Ang II group) further demonstrate the inhibitory effect of Tec on this pathway (Supplementary Table S3). Both *in vivo* and *in vitro* experiments indicate that Tec significantly ameliorates Ang II-induced activation of the STING-NFκB pathway. In summary, Tec inhibits Ang II-induced STING phosphorylation and reduces NFκB pathway activation.

### 3.6 The STING inhibitor H151 eliminates the improvement of tec in cardiac function, left ventricular hypertrophy, and myocardial injury in mice

**FIGURE 6:**
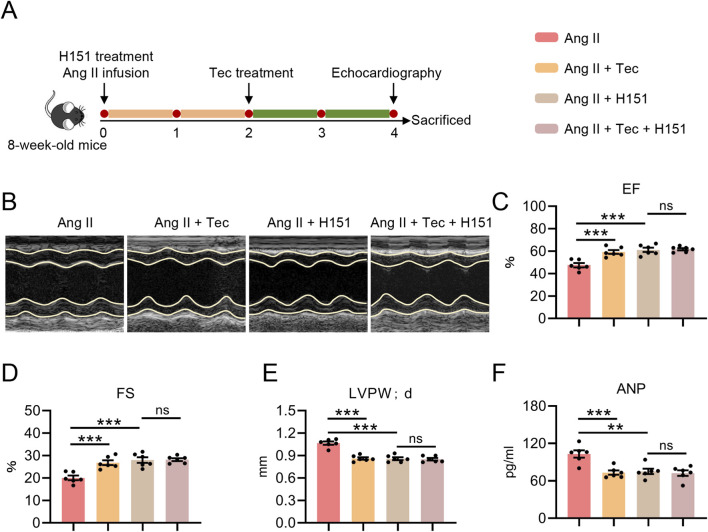
The STING inhibitor H151 eliminates the improvement of Tec in cardiac function, left ventricular hypertrophy, and myocardial injury in mice. **(A)** Schematic diagram of STING inhibitor H151 experimental workflow. Mice received a 4-week infusion of Ang II along with H151 treatment (4 μg/kg i. p.) from the outset, and were additionally treated with Tec (5 mg/kg i. p.) during the final 2 weeks. **(B)** Representative echocardiographic images for each group of mice. **(C)** EF in the indicated groups. **(D)** FS in the indicated groups. **(E)** Left ventricular posterior wall thickness at diastole of mice. **(F)** ANP levels in mice serum. N = 6. ns, p > 0.05. *, p < 0.05. **, p < 0.01. ***, p < 0.001.

To further clarify the role of STING in Tec-mediated treatment of pathological cardiac hypertrophy, as depicted in Figure 6A, we administered the STING inhibitor H151 to mice. Initially, cardiac function was assessed in each group. Consistent with prior findings, Tec significantly alleviated Ang II-induced cardiac dysfunction, as evidenced by EF and FS (Figures 6B–D). Yet, compared with the Ang II + H151 group, the Ang II + H151+Tec group showed no significant difference in EF or FS values (p > 0.05) (Figures 6B–D). Interestingly, we observed a similar outcome for LVPW; d. Tec significantly ameliorated Ang II-induced increases in LVPW; d. However, no significant difference in LVPW; d was observed between mice in the H151+Ang II group and those in the H151+Ang II + Tec group (p > 0.05) (Figure 6E). Furthermore, serum samples were collected from all groups and analysed for marker of heart failure and myocardial injury. The results showed that Tec significantly reduced the Ang II-induced elevation in serum ANP (p < 0.05) (Figure 6F). Nevertheless, compared with the H151+Ang II group, the addition of Tec in the H151+Ang II + Tec group no longer significantly improved this parameter in mice (p > 0.05) (Figure 6F). Notably, H151 alone can largely replicate the beneficial effects of Tec on Ang II-induced cardiac function, left ventricular hypertrophy, and myocardial injury in mice. On this basis, the additional administration of Tec does not confer further protective benefits, indicating that the mitigation of Tec (H151+Tec) in Ang II-induced cardiac dysfunction, left ventricular hypertrophy, and myocardial injury depends on STING activation.

### 3.7 The STING inhibitor H151 eliminates the therapeutic effects of Tec on Ang II-induced pathological cardiac hypertrophy, myocardial fibrosis, and myocardial inflammation in mice

**FIGURE 7:**
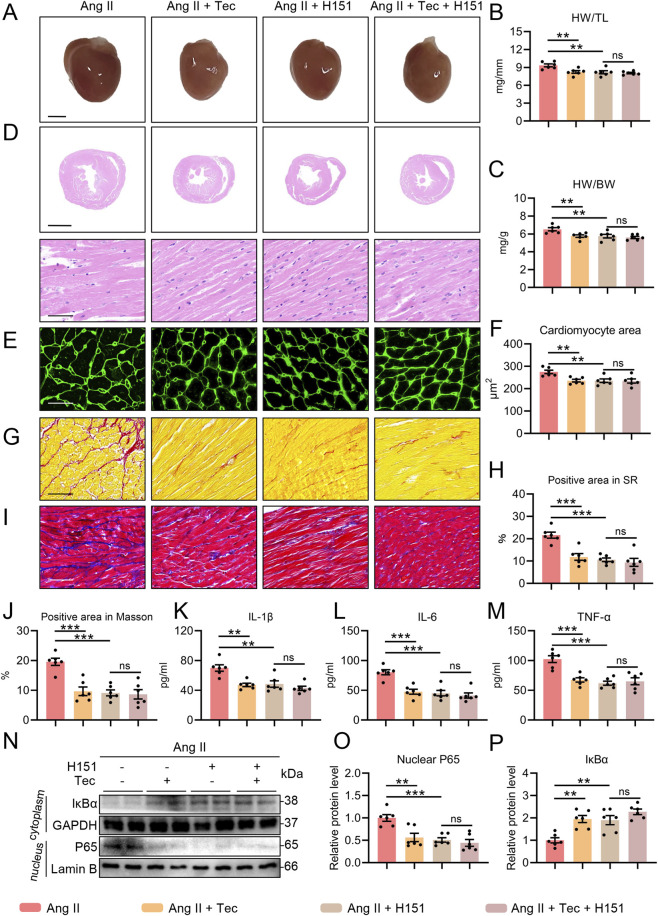
The STING inhibitor H151 eliminates the therapeutic effects of Tec on Ang II-induced pathological cardiac hypertrophy, myocardial fibrosis, and myocardial inflammation in mice. **(A)** Gross image of the heart. Scale bar = 2 mm. **(B)** HW/TL ratio in the indicated groups. **(C)** HW/BW ratio in the indicated groups. **(D)** The representative H&E results under low (Scale bar = 2 mm) and high (Scale bar = 50 μm) magnification. **(E)** The representative image of WGA results. Scale bar = 25 μm. **(F)** Quantitative analysis of panel **(E) (G)** The representative Sirius red staining results. Scale bar = 50 μm. **(H)** Quantitative analysis of panel **(G) (I)** The representative Masson staining results. Scale bar = 50 μm. **(J)** Quantitative analysis of panel **(I) (K)** The level of serum IL-1β. **(L)** The level of serum IL-6. **(M)** The level of serum TNF-α. **(N)** Representative immunoblotting results for the IκBα and nuclear P65 in the heart tissue of mice. **(O)** Quantitative analysis of IκBα. **(P)** Quantitative analysis of nuclear P65. N = 6. ns, p > 0.05. *, p < 0.05. **, p < 0.01. ***, p < 0.001.

Subsequently, we collected hearts from each group of mice for macroscopic examination. The results were consistent with previous findings showing that Tec significantly ameliorated Ang II-induced cardiac enlargement, whereas no significant change in cardiac volume was observed between H151+Ang II group and H151+Ang II + Tec group (Figure 7A). Similar outcomes were observed for the HW/TL ratio and HW/BW ratio (Figures 7B,C). Subsequent H&E and WGA staining of cardiac tissue showed that, consistent with prior findings, Tec treatment significantly alleviated Ang II-induced myocardial fiber thickening and cardiomyocyte cross-sectional area enlargement (p < 0.05). However, We did not observe significant differences in the myocardial fiber and cardiomyocyte cross-sectional area between the H151+Ang II group and the H151+Ang II + Tec group (p > 0.05) (Figures 7E,F). Similar results were also observed in our detection of mRNA levels for Myh7, Anp, and Bnp in myocardial tissue (Supplementary Figure S9A–C). Masson and Sirius red staining further demonstrate that Tec significantly ameliorated Ang II-induced myocardial fibrosis (p < 0.05). Yet, compared with the H151+Ang II group, the H151+Ang II + Tec group showed no further improvement in collagen deposition area (p > 0.05) (Figures 7G–J). Immunohistochemical analysis of Col-1 in myocardial tissue also revealed the aforementioned pathological alterations across all groups (Supplementary Figure S10A,B). Furthermore, we assessed inflammatory levels in serum. ELISA results indicated that Tec significantly suppressed Ang II-induced increases in IL-1β, IL-6, and TNF-α levels. Nevertheless, no significant differences were observed between the H151+Ang II group and the H151+Ang II + Tec group for these markers (p > 0.05). (Figures 7K–M). Similarly, we observed that although Tec significantly reduced the positive area in F4/80 immunohistochemistry, the positive area in the H151+Ang II + Tec group showed no significant change compared to the H151+Ang II group (Supplementary Figure S11A,B). Finally, we detected changes in the NFκB pathway in myocardial tissue. The results of Western blot showed that Tec significantly activates the NFκB pathway (p < 0.05). Furthermore, adding Tec (H151+Ang II + Tec group) to the H151 treatment (H151+Ang II group) does not further inhibit the NFκB pathway (p > 0.05) (Figures 7N–P). Similarly, H151 alone can largely replicate the inhibitory effects that Tec exerts on Ang II-induced pathological myocardial hypertrophy, myocardial fibrosis, myocardial inflammation, and NFκB pathway activation in mice. However, adding Tec on top of this does not provide additional beneficial effects. These results indicate that the inhibitory effects of Tec on Ang II-induced pathological myocardial hypertrophy, myocardial fibrosis, myocardial inflammation, and NFκB pathway activation also depend on STING activation.

### 3.8 STING is crucial for tec in defending against Ang II-induced NFκB pathway activation and cardiomyocyte hypertrophy *in vitro*

To further elucidate the mechanism by which Tec exerts its anti-Ang II-induced cardiac hypertrophy effects, we constructed siRNA targeting STING and applied it to cardiomyocytes, subsequently treating the cells with either Tec or Ang II. Western blot analysis revealed that Tec significantly inhibited activation of the STING/NFκB pathway. However, in STING-knockdown cells, the inhibitory effect of Tec on STING/NFκB pathway activation was no longer significant (Supplementary Figure S12A). Furthermore, we assessed mRNA levels of Myh7, Anp, and Bnp across groups, revealing that Tec significantly suppressed expression of these three cardiac hypertrophy-associated genes. Nevertheless, no significant differences in transcription levels of Myh7, Anp, or Bnp were observed between the Tec + Ang II group and the Tec + Ang II + siSTING group (Supplementary Figure S12B–D). In summary, these findings indicate that STING is crucial for the inhibitory effects of Tec on Ang II-induced NFκB pathway activation and cardiomyocyte hypertrophy *in vitro*.

## 4 Discussion

In this study, we employed Ang II-induced mice as an *in vivo* model to investigate pathological cardiac hypertrophy. We observed that Tec treatment exerted a protective effect against Ang II-induced cardiac hypertrophy, manifested in alleviating cardiac dysfunction, inhibiting myocardial fibrosis, pathological cardiac hypertrophy, and myocardial inflammation. Through SPR, DARTS, and CETSA assays, STING was identified as a binding protein of Tec. Furthermore, *in vivo* and *in vitro* experiments demonstrated that Tec inhibits activation of the STING-NFκB pathway. Utilising the STING inhibitor H151 further confirmed that STING is a critical mediator through which Tec exerts its cardioprotective effects.

Pathological cardiac hypertrophy and heart failure have long been a major global health burden, posing a serious threat to human life and wellbeing. Although cardiac hypertrophy itself represents an adaptive response to mechanical overload or neuroendocrine signalling, failure to control it promptly frequently leads to heart failure, arrhythmias, and ultimately cardiac decompensation (Jibo et al., 2025). Ang II is the most crucial effector within the RAS. Its prolonged action on the heart induces a persistent inflammatory state, which in turn leads to myocardial fibrosis and further exacerbates heart failure (Abhi, Shubham, Ajay, Anupam and Trayambak, 2023). Therefore, suppressing the myocardial inflammatory response is currently one of the key therapeutic strategy for Ang II-induced pathological cardiac hypertrophy (Shiju et al., 2023; Shyam S et al., 2018). Traditional Chinese medicine-derived small-molecule active compounds have garnered significant attention from drug developers due to their low toxicity and readily available raw materials (Yanghao et al., 2022). Our experiments revealed through a series of studies that Tec significantly alleviates Ang II-induced myocardial inflammation. Furthermore, it also improves myocardial fibrosis, pathological hypertrophy, and cardiac dysfunction, confirming its therapeutic potential in Ang II-induced pathological hypertrophy and heart failure. In addition to its significant anti-inflammatory activity, Tec also exhibits antioxidant, anticancer, anti-osteoporotic, and metabolism-modulating properties. Tec reduces cellular reactive oxygen species by activating the Nrf2/HO-1 pathway, thereby inhibiting apoptosis and ferroptosis, ultimately mitigating high-glucose-induced damage to retinal pigment epithelial cells (Yuan, Chengsen, Bingbing, Wei and Shuang, 2025). Additionally, Hong et al. showed that Tec exerts its anticancer effects in NSCLC cells by inhibiting STAT3 phosphorylation, thereby activating DR3 and Fas expression (Saet-Byul et al., 2014). Another research reported that Tec inhibits osteoclast differentiation induced by receptor activator of nuclear factor-κB ligand and macrophage colony-stimulating factor, thereby treating osteoporosis (Yanqiu et al., 2020). Moreover, Tec alleviates type 2 diabetes in mice by modulating IRβ to enhance glucose uptake in adipose tissue and skeletal muscle while inhibiting hepatic gluconeogenesis (Jianfeng et al., 2023). Given its anti-hypertrophic effect identified in our study, together with the multiple beneficial activities of Tec previously reported, we consider Tec a promising multi-functional drug worthy of further development. Future animal and preclinical studies should investigate its efficacy in other disease models to broaden its therapeutic potential.

STING is a key regulatory protein in innate immunity and has long been the subject of considerable attention due to its involvement in the regulation of various cancers (Ali H et al., 2021; Qi et al., 2026; Zhiming et al., 2026). However, an increasing number of researchers have recently discovered its significant role in regulating inflammatory diseases (Ming et al., 2022; Wenqing et al., 2025). Interestingly, the NFκB pathway, recognised as one of the most classic regulatory axis of inflammation, has been reported to be modulated by STING. Within the STING/NFκB pathway, STING recruits IKKβ, thereby promoting the degradation of IκBα and facilitating the nuclear translocation of P65, which in turn activates the expression of downstream inflammatory factors (Meng et al., 2022; Si, Yajuan, Yanpu, Jiaming and Xiao-Fang, 2022). Most importantly, in recent years, a substantial body of research has reported the pivotal role of the STING/NFκB pathway in regulating pathological cardiac hypertrophy. Researchers reported that activation of the STING-NFκB pathway directly induces pathological hypertrophy and heart failure (Lintao et al., 2023). Notably, pharmacological inhibition of STING significantly attenuated NFκB-mediated inflammation, rather than the TBK1-related pathway, leading to marked improvement in cardiac pathology (Lintao et al., 2023). Furthermore, it has been reported that the deubiquitinase OTUD6a drives cardiac inflammation and hypertrophy by stabilizing STING protein, thereby activating downstream NFκB signaling and inflammatory gene expression (Zimin et al., 2024). Of particular interest, recent studies have explored the use of natural compounds derived from traditional Chinese medicine that possess STING-inhibitory activity for treating pathological hypertrophy. Guo et al. demonstrated that chlorogenic acid targets and inhibits STING, thereby blocking NFκB activation and macrophage recruitment, ultimately alleviating pressure overload-induced heart failure and myocardial fibrosis (Kai et al., 2025). Given the above reported research findings, targeting STING and modulating its downstream NFκB pathway holds promise as a potential therapeutic strategy for pathological cardiac hypertrophy. Interestingly, our research has discovered that Tec can directly bind to STING, with this interaction primarily involving the serine residue at position 161 of STING. The Tec-STING interaction inhibits STING phosphorylation, thereby suppressing Ang II-induced excessive activation of the STING-NFκB pathway. This suggests that Tec may function as a novel STING inhibitor, exerting anti-inflammatory effects in the treatment of Ang II-induced pathological cardiac hypertrophy.

Admittedly, although we demonstrated through SPR, DARTS, and CETSA that Tec can directly bind to STING, and confirmed the pivotal role of STING in Tec treatment of Ang II-induced cardiac hypertrophy using the STING inhibitor H151 and siRNA targeted STING, Tec is a small-molecule drug with potential multiple targets. We cannot entirely rule out the possibility that Tec may bind to other target proteins actions upstream or in parallel to STING, which would require further confirmation through knockout mice for STING and other genes. Secondly, we found that Tec significantly suppressed Ang II-induced macrophage infiltration and fibroblast activation, as shown by reduced collagen deposition and Col-1 expression. However, the specific regulatory mechanisms through which Tec modulates distinct subpopulations of macrophages and fibroblasts, as well as their interactions, remain to be fully elucidated. Therefore, future studies should employ techniques such as single-cell RNA sequencing to profile immune infiltrating cells and activated fibroblasts within the tissue, thereby providing deeper insights into the therapeutic mechanisms of Tec in pathological cardiac hypertrophy. Moreover, although Tec has not been found to possess potential toxicity, given the unknown human exposure-response relationship, potential off-target effects, differences in STING between rodents and humans, and the necessity for further medicinal chemistry optimization of Tec, it is essential to conduct pharmacokinetic, pharmacodynamic, and bioavailability studies, as well as long-term safety tests, before broader application of Tec. Validation should also be performed in mice with diverse characteristics (such as female mice and aged mice) and in additional heart failure models (pressure overload models, metabolism-related heart failure, or heart failure with preserved ejection fraction). Ultimately, the interaction between Tec and STING should be examined in human cardiomyocytes or cardiac tissues, such as those derived from induced pluripotent stem cell (iPSC).

## 5 Conclusion

In summary, we demonstrated that Tec significantly ameliorates Ang II-induced myocardial inflammation, fibrosis, and pathological hypertrophy. Transcriptome sequencing suggests Tec treatment may downregulate the STING/NFκB signalling pathway. Mechanically, we further confirmed that Tec directly interacts with STING. In both *in vivo* and *in vitro* experiments, it was demonstrated that Tec inhibits STING phosphorylation and the activation of the downstream NFκB pathway. Our findings indicate that Tec may emerge as a potential lead compound targeting STING for the treatment of Ang II-induced pathological cardiac hypertrophy and hypertensive heart failure. However, prior to clinical application, further studies on the pharmacokinetics, safety, and translational medicine of Tec are required.

## Data Availability

All the data in this study are available upon reasonable request from the corresponding author. The transcriptomic datasets have been deposited in the Gene Expression Omnibus (GEO) database (accession code: GSE327509).
