# Supplementary material for: Tectochrysin alleviates Ang II-induced pathological cardiac hypertrophy by binding to STING and inhibiting STING/NFκB-mediated inflammation
Source: Front Pharmacol. 2026 Apr 23;17:1794994. doi: 10.3389/fphar.2026.1794994 (PMC13149437; doi:10.3389/fphar.2026.1794994)
Supplement: Supplementary file 1 [file Supplementaryfile1.docx]

***Supplementary information***

**Tectochrysin alleviates pathological cardiac hypertrophy by binding to STING and inhibiting STING/NFκB-mediated inflammation**

**Contents in** **Supplementary Files**

Online Supplementary Table S1-3

Online Supplementary Figure S1-12

**Supplementary Table S1. qPCR primer sequences.**

| Gene | Species | Sequence (5' to 3') |
| --- | --- | --- |
| Myh7 | Rat | CCAGAACACCAGCCTCATCAACC  CACCGCCTCCTCCACCTCTG |
| Anp | Rat | GAGCGAGCAGACCGATGAAGC  TCCATCTCTCTGAGACGGGTTGAC |
| Bnp | Rat | CTCCAGAACAATCCACGATG  ACAGCCCAAGCGACTGACT |
| Actb | Rat | AAGTCCCTCACCCTCCCAAAAG  AAGCAATGCTGTCACCTTCCC |
| Myh7 | Mouse | CAGAACACCAGCCTCATCAACCAG  CACAGCTTCTCTTTGATGTCAC |
| Anp | Mouse | AAGAACCTGCTAGACCACCTGGAG  TGCTTCCTCAGTCTGCTCACTCAG |
| Bnp | Mouse | TGCTGGAGCTGATAAGAGAAAA  GAAGGACTCTTTTTGGGTGTTC |
| Actb | Mouse | CTACCTCATGAAGATCCTGACC  CACAGCTTCTCTTTGATGTCAC |

**Supplementary Table S2. The top 10 pathways with the most significant changes in GSEA enrichment analysis.**

| Pathway name | Adjusted-p value |
| --- | --- |
| nod like receptor signaling pathway | 0.009496726 |
| cytosolic dna sensing pathway | 0.009898247 |
| chemokine signaling pathway | 0.009915994 |
| toll like receptor signaling pathway | 0.010066987 |
| c type lectin receptor signaling pathway | 0.011559016 |
| cgas sting signaling pathway | 0.011597938 |
| nf kappa b signaling pathway | 0.014644746 |
| tnf signaling pathway | 0.014828743 |
| rig i like receptor signaling pathway | 0.024152486 |
| b cell receptor signaling pathway | 0.024431063 |

**Supplementary Table S3. Expression changes in representative genes of STING/NFκB signaling pathway.**

| Gene name | Log2 (fold change) |
| --- | --- |
| Igtp | -0.399408863 |
| Irgm1 | -0.479445973 |
| Irgm2 | -0.576391161 |
| Il1b | -0.822057608 |
| Tnf | -1.023938569 |
| Col1a1 | -3.561712398 |
| Col3a1 | -3.342728228 |
| Myh7 | -0.728980164 |
| Nppa | -1.757346681 |
| Nppb | -1.100671233 |
| Aurkb | -2.530275378 |
| Relb | -0.406140591 |
| Tnfsf14 | -2.548678383 |
| Nfkb2 | -0.412898131 |
| Tnfrsf1a | -0.521453046 |
| Tradd | -0.920010781 |
| Icam1 | -0.253749667 |
| Ticam2 | -1.344593518 |
| Ccl21a | -0.933299343 |
| Ccl21b | -0.533250292 |


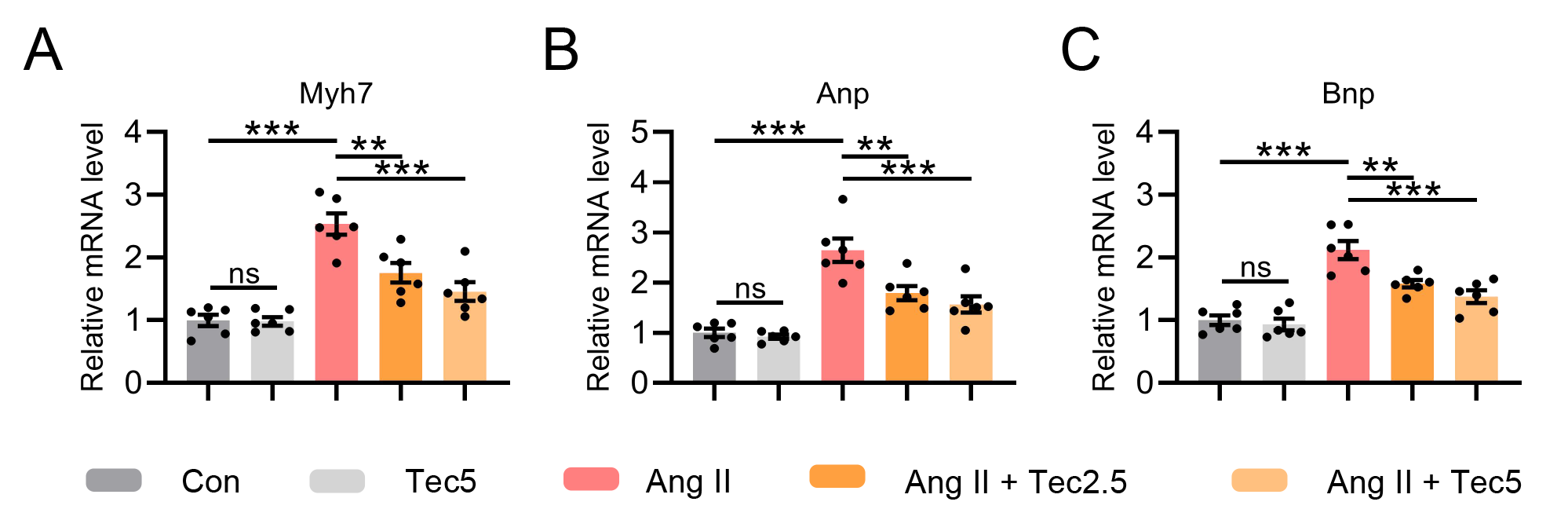


**Supplementary Figure S1. Tec significantly alleviates Ang II-induced pathological cardiac hypertrophy.** (**A**) The relative mRNA level of Myh7. (**B**) The relative mRNA level of Anp. (**C**) The relative mRNA level of Bnp. N=6. ns, p > 0.05. *, p < 0.05. **, p < 0.01. ***, p < 0.001.


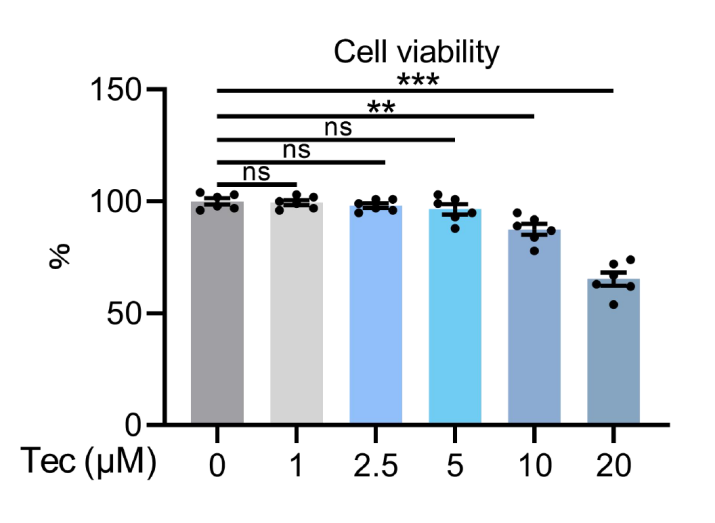


**Supplementary Figure S2. The effect of different concentrations of Tec on cardiomyocyte survival rate.** Cardiomyocytes were treated with different concentrations of Tec for 48 hours, followed by CCK-8 assay to determine cell viability. N=6. ns, p > 0.05. *, p < 0.05. **, p < 0.01. ***, p < 0.001.


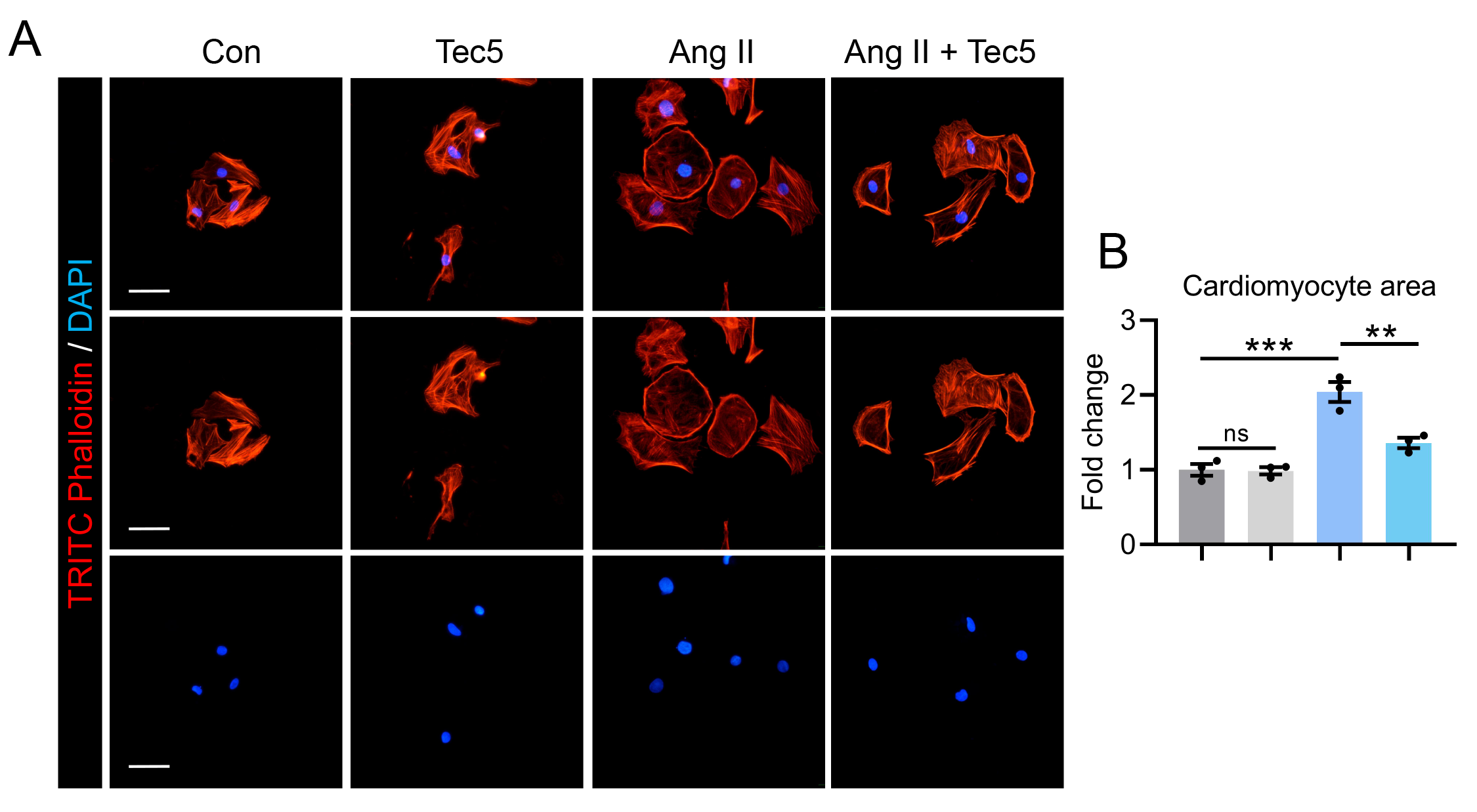


**Supplementary Figure S3. The effect of Tec on Ang II-induced cardiomyocyte hypertrophy.** After pretreatment with Tec or vehicle for 1 h, the primary cardiomyocytes were stimulated with Ang II for 48 h. (**A**) Representative image of phalloidin staining. (**B**) Quantitative analysis of cardiomyocyte area. Scale bar=50 μM. N=3. ns, p > 0.05. *, p < 0.05. **, p < 0.01. ***, p < 0.001.


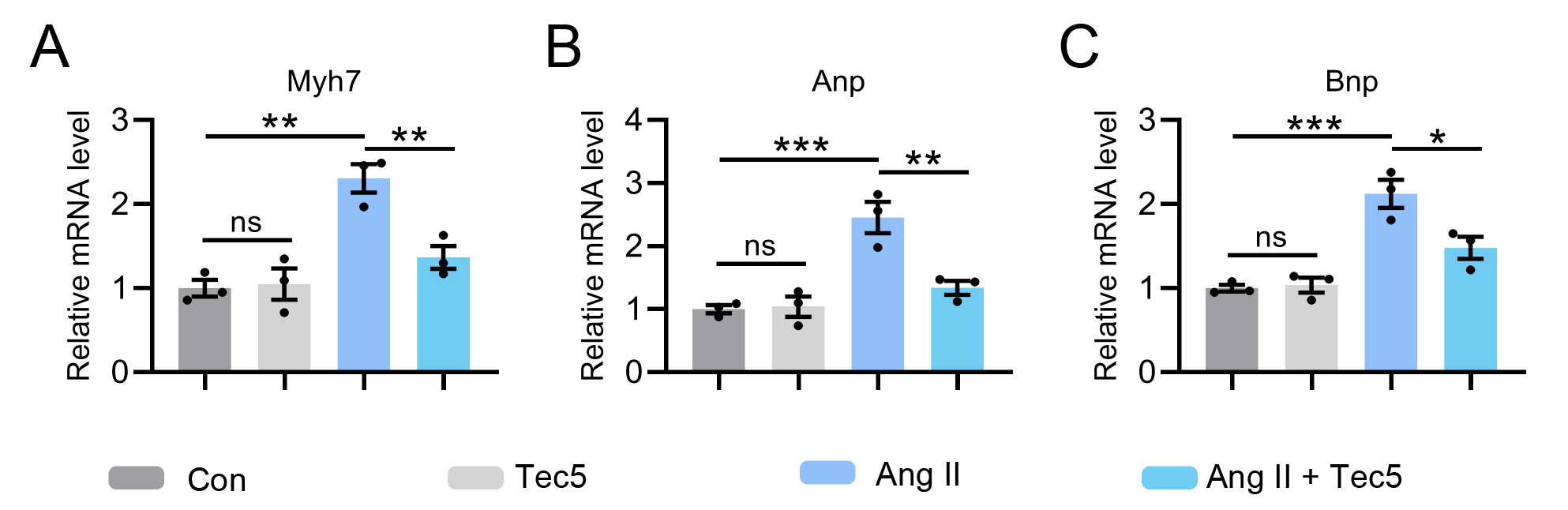


**Supplementary Figure S4. Tec treatment alleviates Ang II-induced cardiomyocyte hypertrophy.** (**A**) The relative mRNA level of Myh7. (**B**) The relative mRNA level of Anp. (**C**) The relative mRNA level of Bnp. N=3. ns, p > 0.05. *, p < 0.05. **, p < 0.01. ***, p < 0.001.


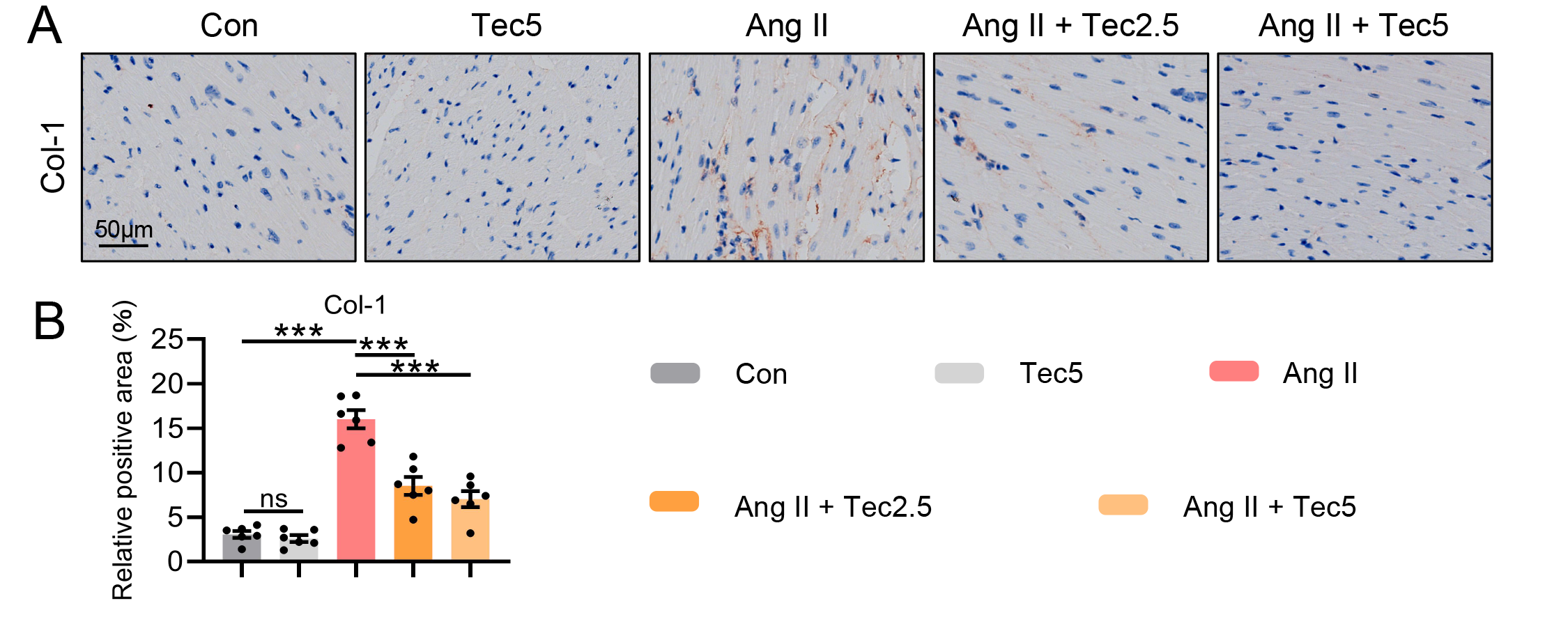


**Supplementary Figure S5. Tec significantly alleviates Ang II-induced myocardial fibrosis.** (**A**) Representative immunohistochemical image of Col-1 in myocardial tissue. (**B**) Quantitative analysis of positive area.N=6. ns, p > 0.05. *, p < 0.05. **, p < 0.01. ***, p < 0.001.


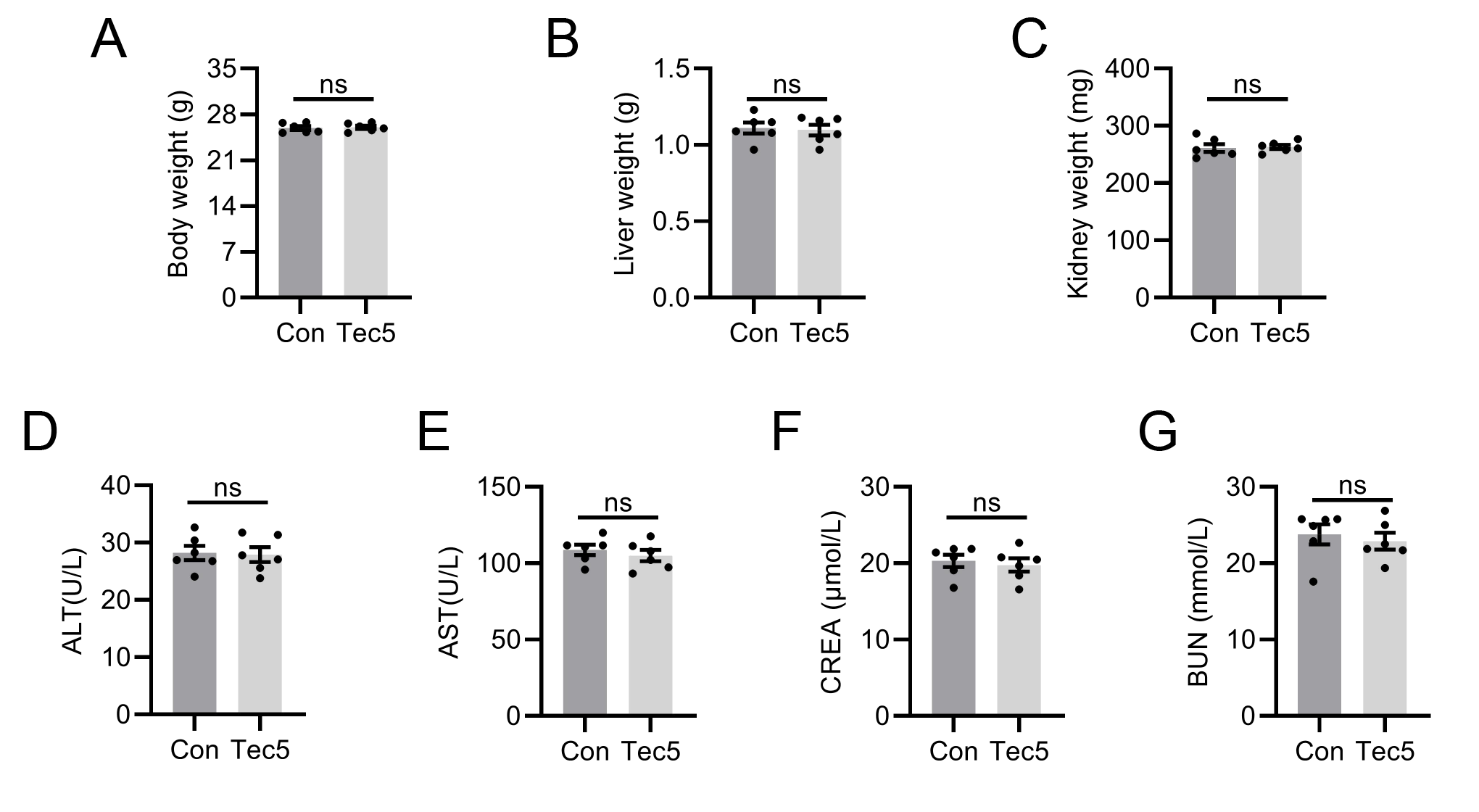


**Supplementary Figure S6. Safety assessment of 5μM Tec treatment.** (**A**) Body weight of the indicated groups. (B) Liver weight of the indicated groups. (C) Kidney weight of the indicated groups. (D) The serum ALT level of the indicated groups. (E) The serum AST level of the indicated groups. (**F**) The creatinine level of the indicated groups. (G) The BUN level of the indicated groups. N=6. ns, p > 0.05.

**
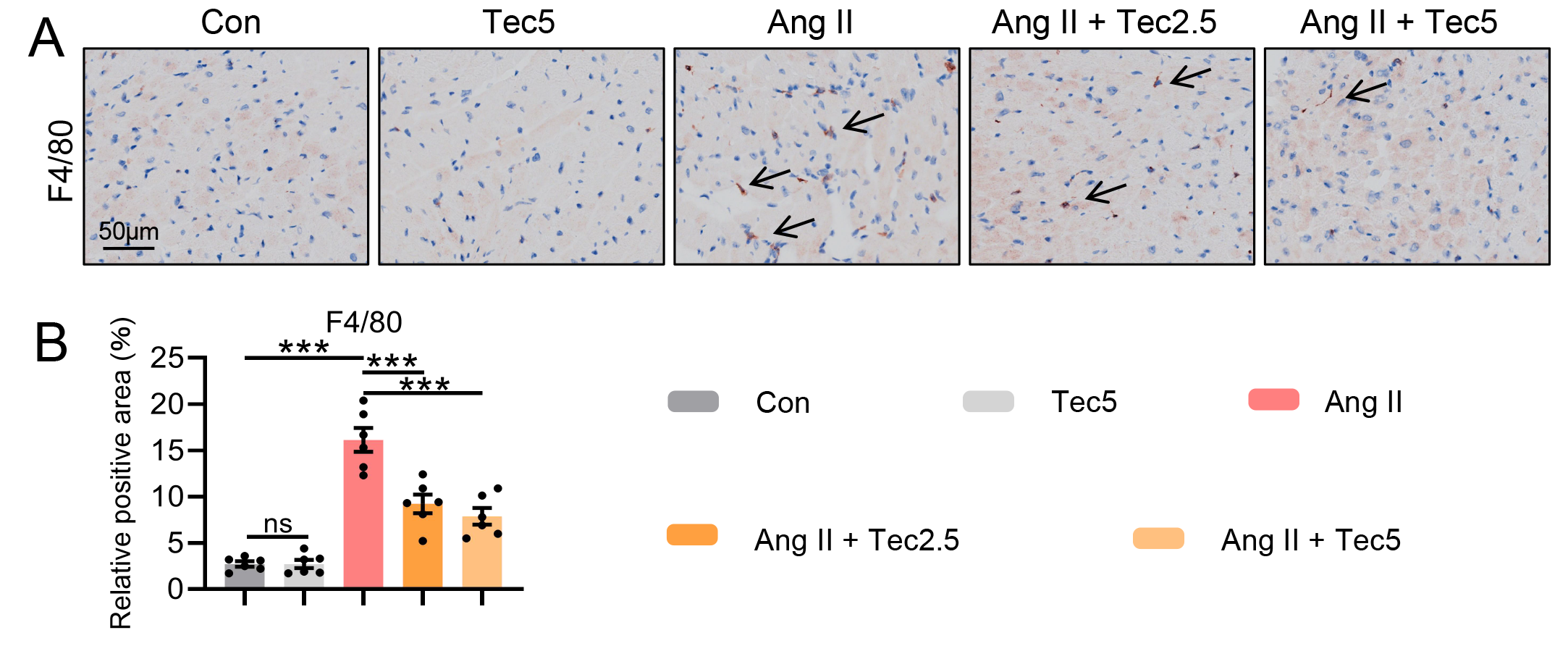
Supplementary Figure S7. Tec significantly alleviates Ang II-induced macrophage infiltration.**  (**A**) Representative immunohistochemical image of F4/80 in myocardial tissue. (**B**) Quantitative analysis of positive area. N=6. ns, p > 0.05. *, p < 0.05. **, p < 0.01. ***, p < 0.001.


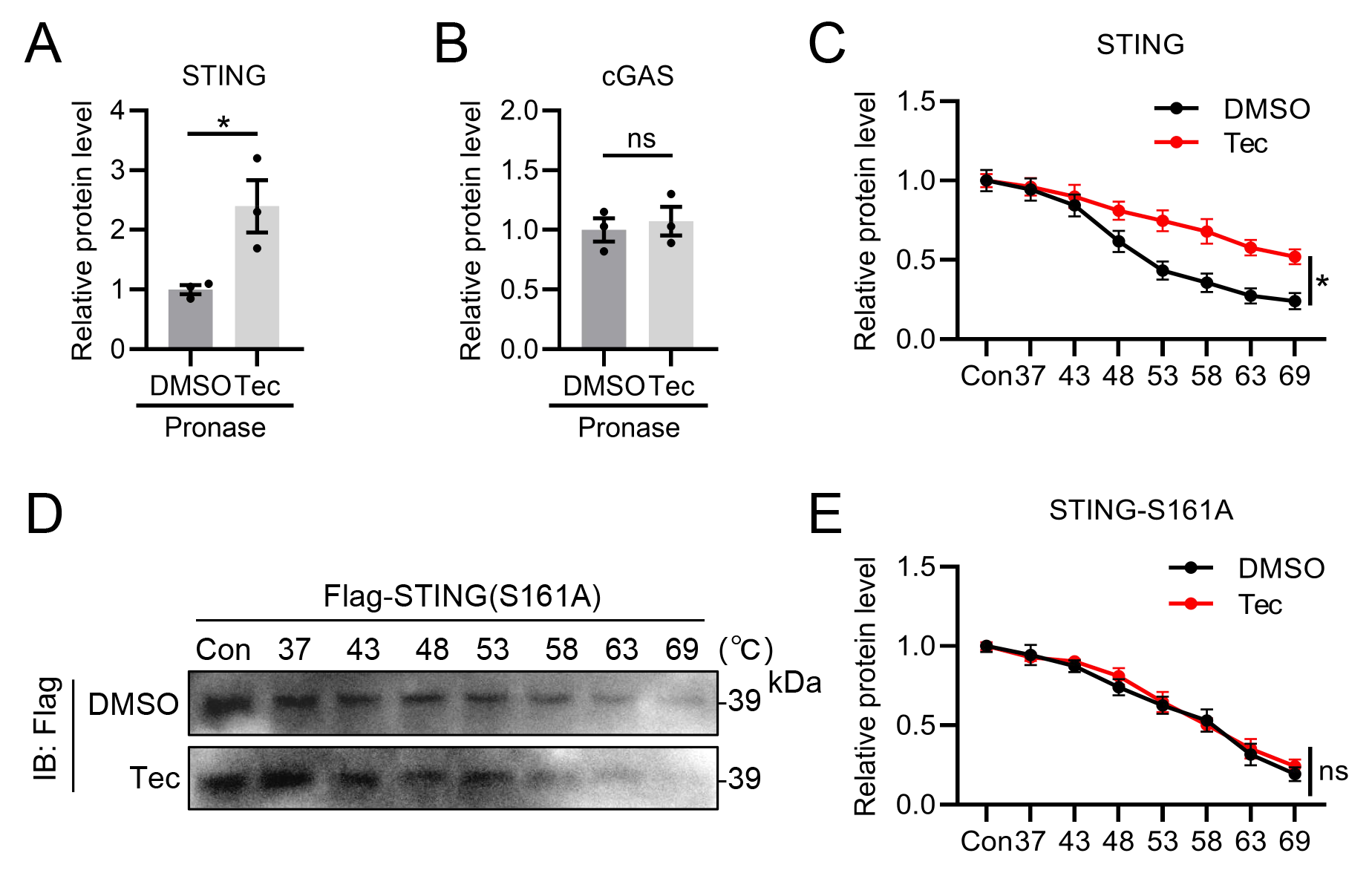


**Supplementary Figure S8. Tec directly binds to STING through Serine 161 of STING.** (**A**) Quantitative analysis of STING bands following treatment with DMSO and Tec under pronase conditions. (**B**) Quantitative analysis of cGAS bands following treatment with DMSO and Tec under pronase conditions. (**C**) Quantitative analysis of STING bands following treatment with DMSO and Tec under heat conditions. (**D**) Representative bands of STING-S161A bands following treatment with DMSO and Tec under heat conditions. (**E**) Quantitative analysis of panel D. N=3. ns, p > 0.05. *, p < 0.05. **, p < 0.01. ***, p < 0.001.


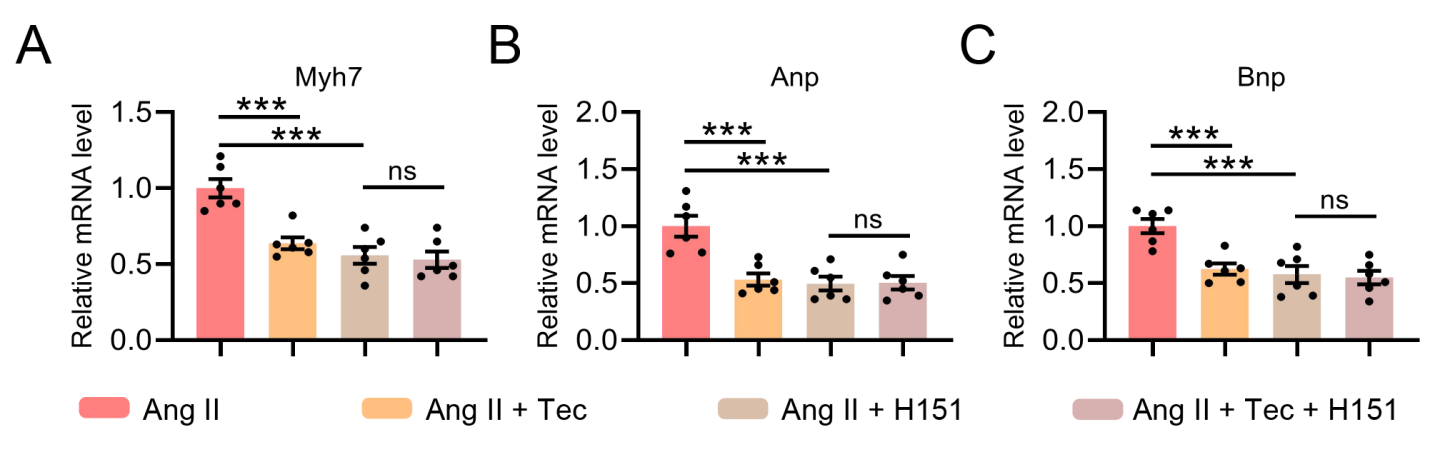


**Supplementary Figure S9. The inhibitory effect of Tec on Ang II-induced pathological cardiac hypertrophy depends on STING inhibition.** (**A**) The relative mRNA level of Myh7. (**B**) The relative mRNA level of Anp. (**C**) The relative mRNA level of Bnp. N=6. ns, p > 0.05. *, p < 0.05. **, p < 0.01. ***, p < 0.001.


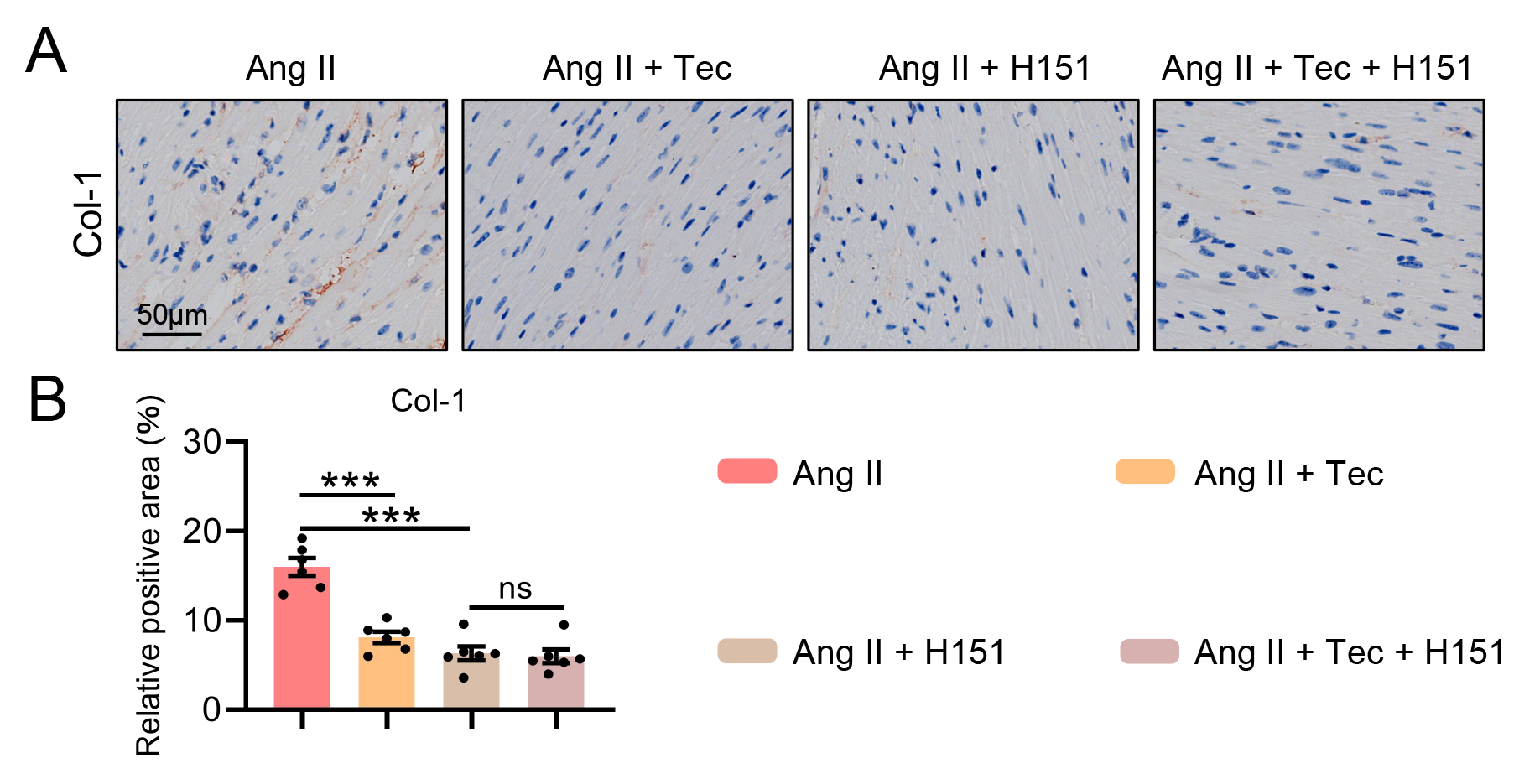


**Supplementary Figure S10. The inhibitory effect of Tec on Ang II-induced myocardial fibrosis was dependent on STING inhibition.** (**A**) Representative immunohistochemical image of Col-1 in myocardial tissue. (**B**) Quantitative analysis of positive area. N=6. ns, p > 0.05. *, p < 0.05. **, p < 0.01. ***, p < 0.001.

**
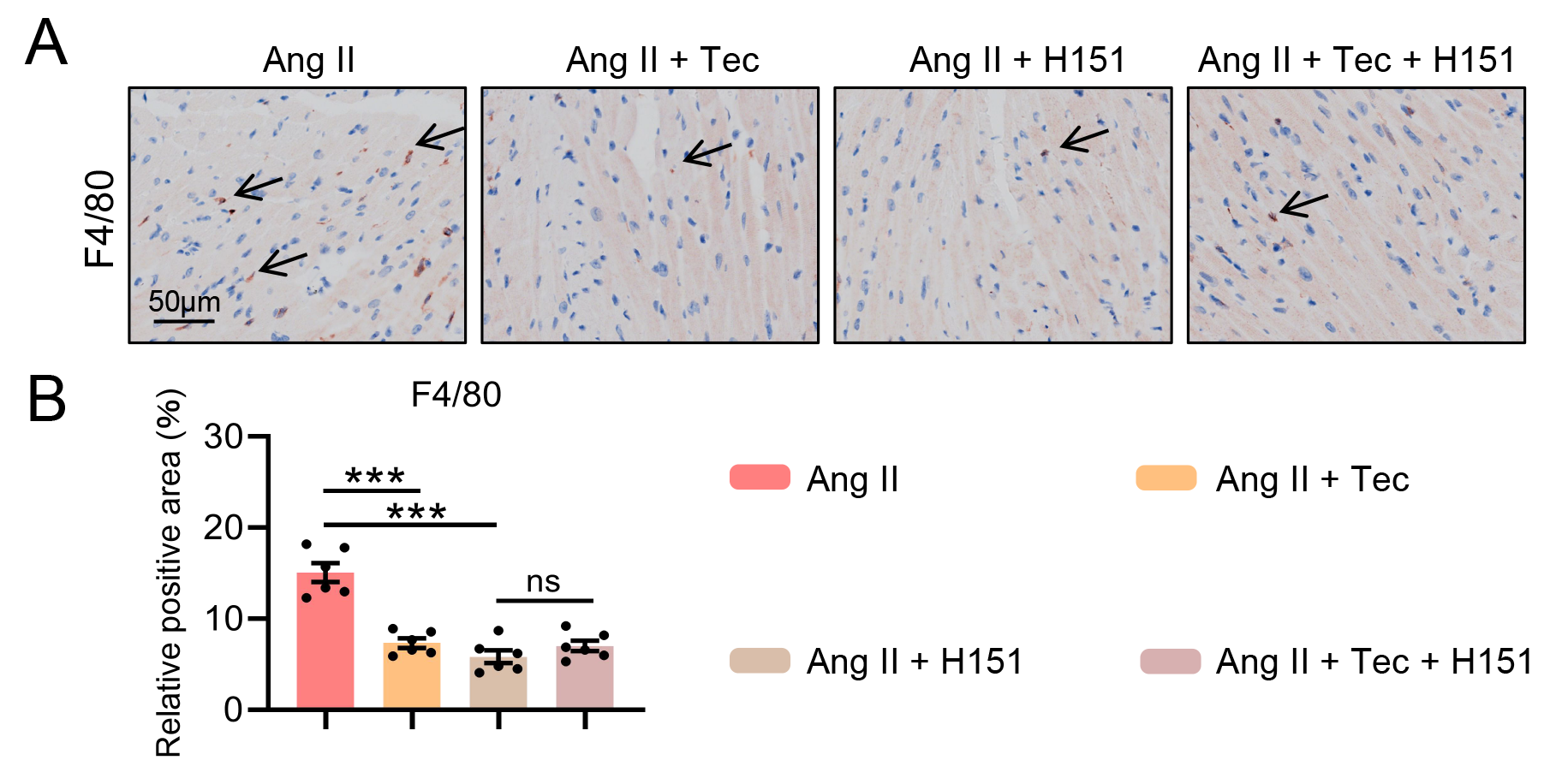
**

**Supplementary Figure S11. The inhibitory effect of Tec on Ang II-induced myocardial macrophage infiltration depends on STING suppression.** (**A**) Representative immunohistochemical image of F4/80 in myocardial tissue. (**B**) Quantitative analysis of positive area. N=6. ns, p > 0.05. *, p < 0.05. **, p < 0.01. ***, p < 0.001.


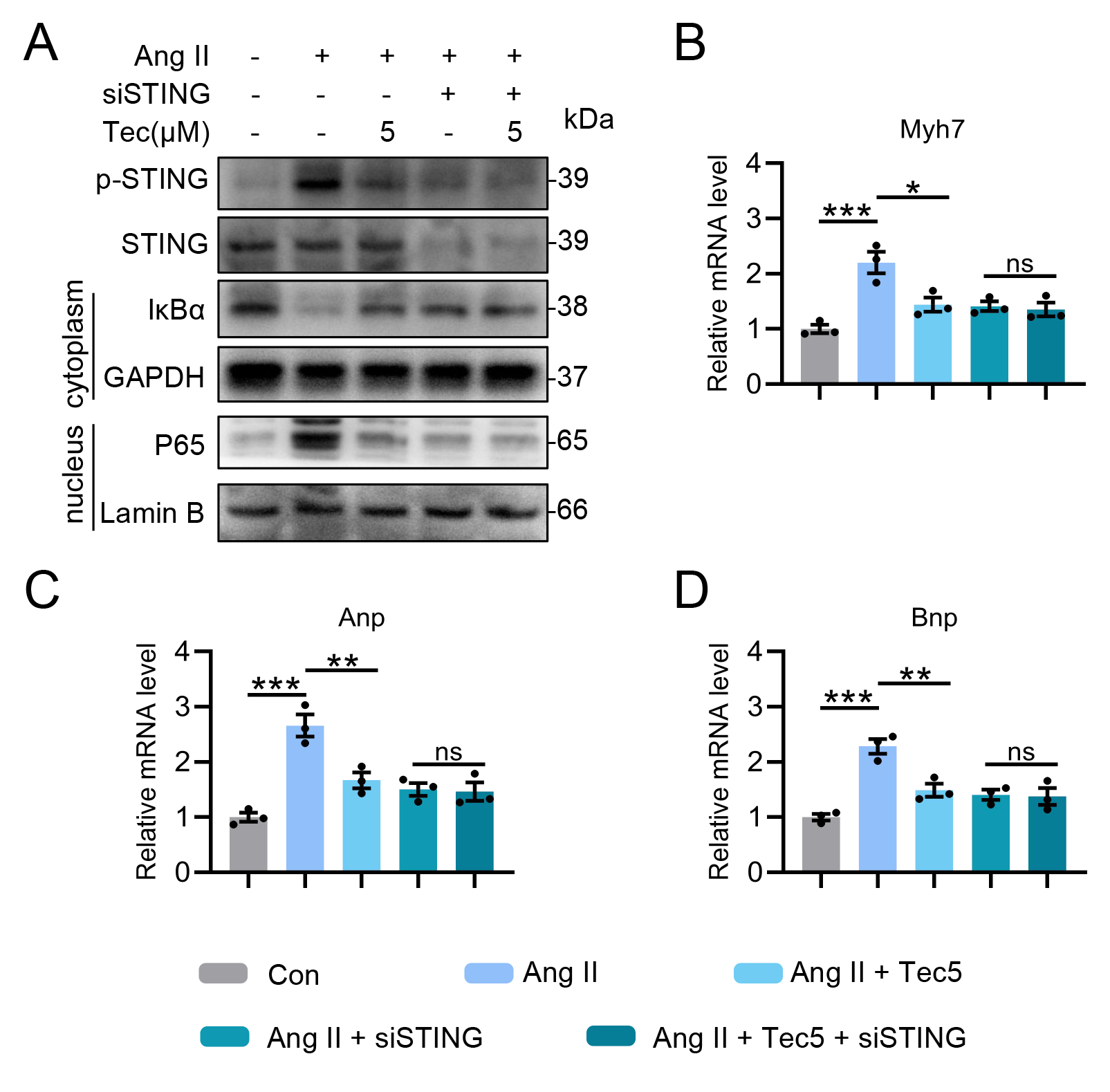


**Supplementary Figure S12. The inhibitory effect of Tec on Ang II-induced cardiomyocyte hypertrophy and the NFκB pathway is dependent on STING.** (**A**) The cardiomyocytes transfected with STING siRNA for 24 h were pretreated with 5 μM Tec and then stimulated with 1 μM Ang II for 48 h. Representative immunoblotting results for the STING/NFκB pathway. (**B**) The relative mRNA level of Myh7. (**C**) The relative mRNA level of Anp. (**D**) The relative mRNA level of Bnp. N=3. ns, p > 0.05. *, p < 0.05. **, p < 0.01. ***, p < 0.001.
